# Shared Negative Experiences Lead to Identity Fusion via Personal Reflection

**DOI:** 10.1371/journal.pone.0145611

**Published:** 2015-12-23

**Authors:** Jonathan Jong, Harvey Whitehouse, Christopher Kavanagh, Justin Lane

**Affiliations:** 1 Coventry University, Coventry, United Kingdom; 2 Institute of Cognitive and Evolutionary Anthropology, University of Oxford, Oxford, United Kingdom; Harvard Medical School, UNITED STATES

## Abstract

Across three studies, we examined the role of shared negative experiences in the formation of strong social bonds—identity fusion—previously associated with individuals' willingness to self-sacrifice for the sake of their groups. Studies 1 and 2 were correlational studies conducted on two different populations. In Study 1, we found that the extent to which Northern Irish Republicans and Unionists experienced shared negative experiences was associated with levels of identity fusion, and that this relationship was mediated by their reflection on these experiences. In Study 2, we replicated this finding among Bostonians, looking at their experiences of the 2013 Boston Marathon Bombings. These correlational studies provide initial evidence for the plausibility of our causal model; however, an experiment was required for a more direct test. Thus, in Study 3, we experimentally manipulated the salience of the Boston Marathon Bombings, and found that this increased state levels of identity fusion among those who experienced it negatively. Taken together, these three studies provide evidence that shared negative experience leads to identity fusion, and that this process involves personal reflection.

## Introduction

Social psychologists have long been puzzled and intrigued by extreme behaviours on behalf of ingroups, from the moral compromise of Nazi *Schutzstaffel* soldiers to the literal self-sacrifice of Japanese *kamikaze* warriors and, more recently, suicide bombers from fundamentalist Islamist groups. Previous research on intergroup conflict have emphasised the role of social identification and deindividuation, positing that under certain circumstances, the activation of an individual’s *social* identity leads to the deactivation of her *personal* identity: this enables the individual to prioritise the group’s values and interests over her own [1–2]. More recent research has implicated another form of group alignment as an important predictor of individual group members’ willingness to self-sacrifice for their groups: identity fusion.

Identity fusion is characterised by a visceral feeling of oneness with the group, such that the borders between one’s personal and social selves are porous [3–4], and the individual’s self-concept and group concept overlap [5]. Identity fusion thus drives costly pro-group behaviour precisely because it blurs the distinction between the self and the group, and thus the distinction between pro-*group* and pro-*self* behaviour. Accordingly, previous research has shown that highly fused individuals are more willing to endorse self-sacrificial pro-group behaviours [4,6], even when controlling for the kinds of group alignment associated with deindividuation [4].

While there is increasing evidence for the role of identity fusion in intergroup behaviour, its causes are still relatively unknown. Synthesizing anthropological and psychological research, Whitehouse and Lanman [7] suggest that intense shared experiences, and negative experiences in particular, may play an important role in the development of identity fusion. They observe, for example, that the ethnographic record is replete with *rites of terror*—highly dysphoric rituals that serve to bind group members together—from ritualized penis bleeding among the Ilahita Arapesh in Papua New Guinea to more culturally familiar forms of hazing in college fraternities. Recent field experiments also support these observations, showing that Mauritian Hindus were more charitable to their groups after participating in Thaipusam rituals involving body piercing, bearing heavy bamboo structures, and pulling carts attached by hooks to participants’ flesh [8]. The psychological literature on self-concept formation similarly suggests that our personal self-concepts are constructed around transformative autobiographical episodes [9–14]. While such self-defining experiences can be positive or negative, there seems to be a unique role for negative experiences, partly because such experiences engender reflective processes (e.g., narrative reflection) in order to be assimilated into the self-concept [15–20]. To the extent that these experiences are shared, Whitehouse and Lanman [7] hypothesize that they lead to the inclusion of the group into individuals’ self concepts, leading to the enduring porosity between personal and social selves that defines identity fusion.

A recent survey of revolutionaries in the aftermath of the 2011 Libyan revolution lends some support to this hypothesis [21]. The survey included front line armed fighters as well as non-fighters, such as workers who serviced vehicles or drove ambulances. When asked to choose between family and battalion as the group they were most fused with, frontline fighters were more likely than non-fighters to choose their battalions over their families. In other words, individuals who had more direct experience of shared negative experience during the course of the revolution were more fused with their comrades.

The present series of studies aims to further investigate the role of shared negative experiences in identity fusion, and to provide preliminary evidence for Whitehouse and Lanman’s [7] hypothesis that shared negative experiences lead to identity fusion via personal reflection. In Study 1, we asked groups on both sides of the sectarian divide in Northern Ireland (Republican v. Unionist) about (a) their negative traumatic or negative experiences as Republicans/Unionists and (b) the extent to which they reflected on these experiences, before measuring their levels of fusion with their communities. In Study 2, we measured similar variables, but this time among victims of a single event, the Boston Marathon Bombing of 2013. In Study 3, we carried out an experimental manipulation to see whether priming memories of the Boston Bombing increased *state* levels of identity fusion.

## Methods and Results

### Study 1

#### Participants

Participants were recruited via the Qualtrics Panel service. Seventy-one women and one hundred and twenty-three men of Irish or British nationality over the age of 40 years (*M*_age_ = 53.95, *SD* = 9.83) and currently residing in Northern Ireland completed the study. During the screening phase, participants were asked whether they identified as “Unionist”, “Republican”, “Don’t Know”, or “Other”. Only participants who identified as “Republican” or “Unionist” were allowed to participate in the study. There were 93 Republicans and 101 Unionists in our sample. Republicans and Unionists were presented with slightly different versions of the study, with the appropriate target groups (Unionist, Republican) assigned in the question texts. We aimed to collect data from 200 individuals (i.e., 100 Unionists, 100 Republicans), basing the sample size on previous experience with similar research: six participants were excluded because they provided incomplete data.

#### Procedure

After providing informed consent, participants completed a sociodemographic questionnaire before proceeding to the study itself, which consisted of three sets of measures. In the first block, participants were asked—in three different ways—about the extent to which they suffered as a result of their Unionism/Republicanism. First, they were presented with a list of 12 kinds of negative experiences a Unionist/Republican may have undergone as a Unionist/Republican (e.g., physical attack verbal attack, public humiliation, property damage; see supplementary materials). Participants were asked to indicate whether they have experienced each type of incident before. Affirmative responses were coded 1 and negative responses were coded 0; scores we summed to form a single index of direct exposure to shared negative experience. Second, they were asked “Over the course of your life, how frequently would you say you suffered—physically, emotionally, or otherwise—for being a [Republican/Unionist]?”; participants responded on a 5-point scale (“Never”, “Very Rarely”, “Rarely”, “Occasionally”, “Frequently”). Third, they were asked “Over the course of your life, how severely would you say you suffered—physically, emotionally, or otherwise—for being a [Republican/Unionist]?”; participants responded on a 5-point scale (“Not at all”, “Slightly”, “Somewhat”, “Moderately”, “Extremely”). These three measures were treated as separate, albeit related measures of experience of shared negative experience.

In the second block, participants were asked two questions designed to briefly assess the extent to which they have thought or reflected on their negative experiences as Republicans or Unionists. They were asked “How often do you think about these experiences?” (anchored at “I have only thought about them a little bit” and “I have spent many years reflecting on them” on a 6-point scale) and “How much have these experiences been on your mind?” (anchored at “I have thought about them sporadically” and “They are always on my mind” on a 6-point scale). Scores on these two items were averaged together to form a single measure of reflection.

In the third block, participants completed a validated measure of identity fusion, Gomez et al.’s [4] verbal fusion questionnaire on a 6-point scale, anchored at Strongly Disagree and Strongly Agree. The target groups were “Unionism” and “Republicanism” for Unionists and Republicans respectively.

#### Results

Scores on the index of direct exposure to shared negative experiences were low (*M* = 2.87, *SD* = 3.84) and significantly positively skewed (skewness = 1.23, *SE* = .175; kurtosis = .209, *SE* = .347); only 52.6% or participants reported experiencing any of the negative experience listed. Similarly, scores on the measure of frequency of shared negative experience (*M* = 1.4, *SD* = 1.321; skewness = .494, *SE* = .175; kurtosis = −1.046, *SE* = .347) and on the measure of suffering severity (*M* = .59, *SD* = 1.091; skewness = .494, *SE* = .175; kurtosis = −1.046, *SE* = .347) were also positively skewed. As expected, all three measures of shared negative experience were significantly inter-correlated (*r*s = .366–.733, *p* < .01). Cronbach’s alpha indicated good reliability for both the two-item measure of reflection (α = .949) and the seven-item measure of identity fusion (α = .953).

To test the hypothesis that shared negative experience leads to identity fusion via personal reflection, three separate mediation analyses were conducted using ordinary least squares path analysis in Hayes’ PROCESS macro (Model 4) for SPSS [22]. A bias-corrected bootstrap analysis based on 5,000 bootstrap samples were run; such analyses are very robust against violations of normality assumptions [23].

The first analysis examined the hypothesised effects of shared negative experience on identity fusion, using the index of direct exposure to shared negative experience. We found that participants who reported experiencing more negative incidents also spent more time reflecting on their negative experiences as Unionists/Republicans (*a* = .1902, *SE* = .0237, *CI* 95% = .1434–.2371), and participants who spent more time reflecting on their negative experiences were also more fused with their parties (*b* = .2922, *SE* = .0646, *CI* 95% = .1647–.4196). A bias-corrected bootstrap analysis based on 5,000 bootstrap samples also revealed a significant indirect effect (*ab* = .0556, *SE* = .0131, *CI* 95% = .0336–.0850). On this measure of shared negative experience, there was no evidence that direct exposure to shared negative experience influenced identity fusion to the party independent of its effect on reflection (*c*’ = .0325, *SE* = .0245; *CI* 95% = −.0159–.0809).

The second analysis examined the hypothesised effects of frequency of shared negative experience. We found that participants who reported greater frequency of shared negative experience also spent more time reflecting on their negative experiences as Unionists/Republicans (*a* = .6517, *SE* = .0643, *CI* 95% = .5249–.7784), and participants who spent more time reflecting on their negative experiences were more fused with their parties (*b* = .2732, *SE* = .0692, *SE* = .1367–.4097). A bias-corrected bootstrap analysis based on 5,000 bootstrap samples also revealed a significant indirect effect (*ab* = .1780, *SE* = .0479, *CI* 95% = .0943–.2848). There was no evidence that frequency of shared negative experience influenced identity fusion to the party independent of its effect on reflection (*c*’ = .1154, *SE* = .0764; *CI* 95% = −.0352–.2660).

The third analysis examined the hypothesised effects of participants’ subjective evaluations of how much they suffered, physically, emotionally, or otherwise as Unionists/Republicans. We found that participants who reported greater suffering also spent more time reflecting on their negative experiences as Unionists/Republicans (*a* = .5164, *SE* = .0902, *CI* 95% = .3385–.6943), and participants who spent more time reflecting on their negative experiences were more fused with their parties (*b* = .4638, *SE* = .0746, *CI* 95% = .3166–.6110). A bias-corrected bootstrap analysis based on 5,000 bootstrap samples also revealed a significant indirect effect (*ab* = .1228, *SE* = .0359, *CI* 95% = .0614–.2038). There was also a significant direct effect of subjective suffering on fusion independent of its effect on fusion (*c’* = .3410, *SE* = .0776, *CI* 95% = .1879–.4941).

The aim of this study was to test the hypothesis that shared negative experience is a causal antecedent to identity fusion, via personal reflection. Correlational evidence cannot provide direct evidence for causal hypotheses, but a failure to find the predicted correlations would falsify our causal account. Furthermore, path analyses of correlational data can provide initial evidence of the plausibility of a causal model. Using three separate albeit conceptually related and significantly inter-correlated measures of shared negative experience, we found that direct experience of different kinds of group-related negative experience, the frequency of such shared negative experience, and the self-reported degree of suffering as a member of their group each predicted the extent to which participants reflected on their negative experience, which in turn predicted identity fusion with their group.

As with Whitehouse et al.’s [21] study on Libyan revolutionaries, it is impossible to confidently determine the causal direction among the variables in our model. However, as it makes little sense to treat participants’ reflection on their shared negative experience as a causal antecedent of said negative experience, the only alternative models would have identity fusion as the independent variables, shared negative experience as the mediator, and levels of reflection as the dependent variable. That is, it is possible that fusion leads participants to experience more shared negative experience, which then leads to increased reflective activity. While this hypothesis is not theoretically motivated, another three mediation analyses were run to explore the possibility of this alternative model. In each case, there was an indirect effect of identity fusion on reflection via shared negative experience. The indirect effect with the index of direct exposure to shared negative experience as the mediator was .1374 (*SE* = .0429, *CI* 95% = .0640–.2328); the indirect effect with frequency of shared negative experience as the mediator was .1923 (*SE* = .0480, *CI* 95% = .1024–.2925). As these effect sizes are similar to, and at least numerically if not statistically larger than the effect sizes of the theoretically-driven model, the theoretical implications of this first study are somewhat ambiguous. We cannot rule out the possibility that people who are more fused to their groups experience more shared negative experience as a result of their identity fusion, which in turn leads to increased reflective activity on their shared negative experience.

#### Discussion

Given the ambiguity regarding causal direction in Study 1, we ran a second correlational study, in a different context. Rather than a prolonged conflict like the Northern Irish Troubles, where it was likely that highly fused people might deliberately put themselves in danger, thereby increasing their likelihood of suffering shared negative experience, we looked at a one-off negative event: the 2013 Boston Marathon Bombing. The aim of this study was to conceptually replicate the results of Study 1 and to disambiguate the causal direction of the relationships among identity fusion, shared negative experience, and personal reflection.

### Study 2

#### Participants

Participants were recruited via Amazon’s Mechanical Turk website [24]. Forty-two women and seventy-three men (*M*_age_ = 29.3, *SD* = 8.24) who currently live or have previously lived in Boston for a substantial period completed the study; only participants who supplied their previous or current Boston ZIP code were permitted to participate. Each participant was paid US$1 as remuneration. We aimed to collect data from 120 participants based on previous similar research using Amazon Mechanical Turk: five participants were excluded for providing incomplete data.

#### Procedure

Participants were redirected from Amazon’s Mechanical Turk website to an online survey hosted by Qualtrics Survey Software. After providing informed consent, participants completed a sociodemographic questionnaire before proceeding to the study itself. The study consisted of two phases. In the first phase of the study, all participants were asked to “recall as vividly as possible” their experience of the “Boston Marathon Bombings that took place in April 2013”, and to type up their recollections in a space provided. The purpose of this task was to ensure that the correct event—the 2013 Boston Marathon Bombing—was salient in the minds of all participants.

After this, participants completed three clusters of questions. As with Study 1, we were interested in three variables: (a) the extent of participants’ shared negative experience, (b) the extent of participants’ reflection about the event(s), and (c) participants’ fusion with their ingroups. In contrast to the Troubles in Northern Ireland, the 2013 Boston Bombing was a single discrete event. Rather than presenting a checklist and asking participants about the frequency of particular kinds of incidents, we asked participants “Did you see or hear or otherwise experience any aspect of the bombing directly?" and “Were you or any family members or friends directly injured in any way during the bombing?” Affirmative responses were coded 1 and negative responses were coded 0; scores we summed to form a single index of exposure to shared negative experience. As in Study 1, we also asked for participants’ subjective judgements of their own negative experience by asking, “How severely would you say you suffered—physically, emotionally, or otherwise—as a result of the Boston Marathon Bombing?” on a 5-point scale anchored at “Not at all” to “Extremely”.

To measure the extent to which participants reflected about the 2013 Boston Bombing, we modified our previous questions used in Study 1, which only assessed the extent to which participants thought about the event generally. In this study, we asked more specific questions that sought to capture deeper, more actively engaged forms of reflection that include interpretive and counterfactual reasoning. This is in keeping with Whitehouse and Lanman’s [7] assertion that the relevant kind of reflection “generates richer representations of the episode and its significance” (p. 680). We therefore asked participants three questions on 6-point scales anchored at “I have only thought about it a bit” and “It is always on my mind”: “How much do you think about your experience of the Boston Marathon Bombings?”; “How much do you think about the meaning of the Boston Marathon Bombings?”; “How much do you think about how the Boston Marathon Bombings could have turned out differently (e.g., how they could have been prevented, or how they could have been worse)?”. Scores on these three items were averaged together to form a single measure of reflection.

To measure identity fusion with Boston, participants completed Gomez et al.’s [4] verbal fusion questionnaire (with Boston as the target group) on a 6-point scale, anchored at Strongly Disagree and Strongly Agree.

#### Results

As anticipated, scores on the exposure to shared negative experience index were low, and significantly positively skewed (skewness = 2.235, *SE* = .226; kurtosis = 4.251, *SE* = .447); only 23.9% of participants reported being injured or otherwise experiencing the 2013 Boston Bombing directly or had family and friends who were injured during the incident. In contrast, scores on the measure of subjective suffering were normally-distributed (skewness = .173, *SE* = .226; kurtosis = −.503, *SE* = .447). As expected, these two measures of shared negative experience were significantly correlated (*r* = .225, *p* < .05). Cronbach’s alpha indicated good reliability for both the three-item measure of reflection (α = .809) and the seven-item measure of identity fusion (α = .924).

To test the hypothesis that shared negative experience leads to identity fusion via a process of reflection, two mediation analyses were conducted using ordinary least squares path analysis in Hayes’s PROCESS macro (Model 4) for SPSS [22]. Bias-corrected bootstrap analyses based on 5,000 bootstrap samples were run; such analyses are very robust against violations of normality assumptions [23].

The first analysis examined the hypothesised effects of direct exposure to the 2013 Boston Bombings. We found that participants who reported greater direct exposure to the 2013 Boston Bombings also reflected more on their experience (*a* = .5990; *SE* = .2563; *CI* 95% = .0911–1.1069), and participants who reflected more on their experience of the 2013 Boston Bombing were more fused with Boston (*b* = .4535; *SE* = .0776; *CI* 95% = .2997–.6073). A bias-corrected bootstrap analysis based on 5,000 bootstrap samples also revealed a significant indirect effect (*ab* = .2716; *SE* = .1540; *CI* 95% = .0392–.6428). There was no evidence that direct exposure to the bombings influenced identity fusion to Boston independent of its effect on reflection (*c*’ = .2274, *SE* = .2147; *CI* 95% = -.1980–.6529).

The second analysis examined the hypothesised effects of subjective suffering as a result of the 2013 Boston Bombings. We found that participants who reported greater severity of physical, emotional, or other suffering as a result of the incident also reflected more on their experience (*a* = .4397, *SE* = .0974, *CI* 95% = .2468–.6327), and participants who reflected more on their experience were more fused with Boston (*b* = .3840, *SE* = .0796, *CI* 95% = .2264–.5417). A bias-corrected bootstrap analysis based on 5,000 bootstrap samples also revealed a significant indirect effect (*ab* = .1689; *SE* = .0551; *CI* 95% = .0801–.3005). There was also a direct effect of subjective suffering on identity fusion to Boston independent of its effect on reflection (*c*’ = .2456 *SE* = .0892; *CI* 95%—.0689–.4223).

As with Study 1, the aim of this study was to test the hypothesis that shared negative experience is a causal antecedent to identity fusion; we also expected that the process by negative experiences affect identity fusion involves reflecting of the experiences in question. Using two separate albeit conceptually related and significantly inter-correlated measures of shared negative experience, we found that direct exposure to the 2013 Boston Bombings and the self-reported degree of suffering as a result of that event predicted the extent to which participants reflected on their negative experience, which in turn predicted identity fusion with Boston. Thus, we successfully conceptually replicated the simple mediation model found in Study 1.

While the choice of the 2013 Boston Bombing was intended to mitigate the problems faces previously regarding causal direction, it is still possible that people who were fused with Boston were more likely to be present at the site of the incident or to have participated in the marathon, or otherwise more likely to suffer as a result of the 2013 Boston Marathon Bombing. With this in mind, we ran two more mediation analyses to explore the possibility that levels of fusion predict shared negative experience, which in turn predicts reflective activity. In contrast to our theoretically-driven model, there was no indirect effect of fusion on reflection with the index of direct exposure to shared negative experience as the mediator (*ab* = .0257, *SE* = .0235, *CI* 95% = −.0042–.0942). However, with the measure of subjective suffering as the mediator, there was a significant albeit small indirect effect (*ab* = .0973, *SE* = .0431, *CI* 95% = .0246–.1967).

#### Discussion

The results of Study 2 provide some assurance that the predicted causal model is the best interpretation of the data. However, as with any cross-sectional correlational study, it is impossible to be certain of any given causal interpretation: correlation does not entail causation. To answer such causal questions definitively, a longitudinal study is required. However, to bolster the claim that shared negative experience leads to identity fusion, we ran an experiment in the context of the 2013 Boston Marathon Bombing.

If Study 1 and Study 2 examined the role of the *chronic* cognitive accessibility of negative shared experiences—that is, the dispositional salience of the experiences, as indicated by the extent to which individuals think about them—in identity fusion, then Study 3 examines the effect of temporarily increased salience of such experiences on identity fusion. In this study, participants were either primed with the 2013 Boston Marathon Bombing or with a neutral event; they then completed a self-report affect measure; finally, they completed two measures of state levels of identity fusion with Boston. We hypothesised that, consistent with the notion that shared negative experience increased identity fusion, participants in the 2013 Boston Bombing condition would report higher levels of state fusion, and that this effect would be moderated by negative affect.

### Study 3

#### Participants

Participants were recruited via Amazon’s Mechanical Turk website [24]. Forty-one women and fifty-eight men (*M*_age_ = 29.5, *SD* = 7.98) who currently live or have previously lived in Boston for a substantial period completed the study; only participants who supplied their previous or current Boston ZIP code were permitted to participate. Each participant was paid US$1 as remuneration. We aimed to collect data from 100 participants, based on previous similar studies on Mechanical Turk: one participant was excluded for providing incomplete data.

#### Procedure

Participants were redirected from Amazon’s Mechanical Turk website to an online survey hosted by Qualtrics Survey Software. After providing informed consent, participants completed a sociodemographic questionnaire before proceeding to the study itself. The study consisted of three phases. In the first phase of the study, participants were randomly assigned into one of two conditions. In the Boston Bombing condition, participants were asked to recall as vividly as possible “the Boston Marathon Bombings that took place in April 2013”. They were asked, “What happened at the bombing? Where were you when it happened? Did it affect you or anyone you know directly? How did it make you feel?”, and to write down their recollections of the event and their experiences in a space provided. In the control condition, participants were asked to recall as vividly as possible “a recent experience of running errands in Boston, doing commonplace things such as shopping for groceries”. They were asked, “What were the errands in question? Where did you go? Did you accomplish your goals? What was the commute like? How did it make you feel?”, and to write down their recollections of the event and their experiences in a space provided.

In the second phase, participants completed Mehrabian and Russell's [25] semantic differential measure of core affect. The 18-item measure consists of three subscales—valence, arousal, and dominance—that capture three dimensions of core affect. Only the valence subscale was of interest in this study, as it is a measure of positive/negative affect. In the third phase, participants completed two measures of state fusion. First, they were presented with the Dynamic Identity Fusion Index (DIFI; http://www.uned.es/fusion/difi/ [5]), a single-item pictorial measure of identity fusion. The DIFi consists of a dynamic image of a small circle labeled “Me” and a large circle labeled “Boston”; participants are asked to click and drag the “Me” circle toward the “Boston” circle “to the position that best captures their current relationship with [Boston]”. The DIFI produces two outputs: a distance score and an overlap score, of which the latter is the more reliable and construct valid [5]; in this study, the overlap score was used as our first measure of state fusion. Second, they were presented with a state version of Gómez et al.'s [4] 7-item verbal fusion scale; the state version of the measure differs from the standard version only in that participants are asked to rate statements about how they “feel about Boston right now”. As with the standard verbal fusion scale, individual item scores were averaged to produce a mean score for each participant.

#### Results

Mehrabian and Russell’s [25] semantic differential measure of core affect produced three scores, for valence, arousal, and dominance respectively. Consistent with the circumplex model of core affect [26–27], valence and arousal were uncorrelated. Valence was significantly correlated with dominance (*r* = .585, *p* < .001), such that participants who felt more positive also felt more dominant. For inferential statistical analyses valence was reverse-scored, such that higher scores indicated more *negative* affect. Valence is the only subscale of theoretical significance in the present study; arousal and dominance scores were therefore dropped from subsequent analyses. The DIFI produced two scores, for distance and overlap; distance and overlap were significant correlated (*r* = .947, *p* < .001). Scores on the 7-item verbal fusion scale were averaged together to produce a single score of state fusion for each individual. Verbal fusion scores were significantly correlated with both the distance (*r* = .712, *p* < .001) and overlap (*r* = .698, *p* < .001) scores. As Jiménez et al. [5] found that the overlap score is the more reliable and construct valid of the two, the distance score was dropped from subsequent analyses.

As a preliminary analysis, we examined the main effect of the priming manipulation on all the relevant measured variables (i.e., reverse-scored valence, DIFI overlap score, verbal fusion). A one-way ANOVA found a main effect on valence, *F*(1, 97) = 57.606, *p* < .01; however, there were no main effects of the priming manipulation on DIFI and verbal fusion scores. Next, we tested the hypothesis that the effect of increased salience of shared negative experience on fusion is moderated by negative affect. First, a simple moderation model was tested in Hayes’s PROCESS macro (Model 1) for SPSS [22], with priming condition as the independent variable, average verbal fusion score as the outcome variable, and valence as the moderator. Valence was mean-centred for this analysis. Again, there was no main effect of priming condition on identity fusion, *b1* = .1244, *SE =* .1329, *ns*; nor was there a main effect of negative affect on identity fusion, *b*2 = −.0038, *SE* = .0784, *ns*. However, the interaction between priming condition and negative affect had a significant effect on identity fusion, *b3* = .1626, *SE* = .0782, *p <* .05, *CI* 95% = .0074–.3179. The increase in amount of variance explained as a result of the interaction term was also statistically significant, *ΔR* ^*2*^ = .0498, *F*(1, 97) = 5.2492, *p* < .05. Probing this interaction effect further, we used PROCESS to estimate of the effect of priming condition at the mean of the moderator (i.e., neutral affect), and at one standard deviation below the mean (i.e., positive affect) and one standard deviation above the mean (i.e., negative affect). As can be seen in Table 1 below, increased salience of the 2013 Boston Marathon Bombing only increased state levels of identity fusion among participants who reported high negative affect.

**Table 1:** The effect of 2013 Boston Marathon Bomb prime on verbal fusion.

Negative affect	Effect	SE	*p*	LLCI	ULCI
−1.9753	−1.969	.1931	.3104	−.5800	.1863
.0000	.1244	.1329	.3518	−.1394	.3882
1.9753	.4456	.2140	.0400*	.0208	.8704

Similarly, we ran the same analysis with the overlap score from the DIFI as the outcome variable. There was no main effect of priming condition on identity fusion, *b1* = 2.6542, *SE =* 4.57, *ns*; nor was there a main effect of negative affect on identity fusion, *b*2 = −.5153, *SE* = 2.5541, *ns*. However, as with the verbal fusion scores, the interaction between priming condition and negative affect had a significant effect on identity fusion, *b3* = 5.3850, *SE* = 2.5678, *p <* .05, *CI* 95% = .2873–10.4827. The increase in the amount of variance explained as a result of the interaction term was also statistically significant, *ΔR* ^*2*^ = .0431, *F*(1, 95) = 4.398, *p* < .05. As can be seen in Table 2 below, increased salience of the 2013 Boston Marathon Bombing only increased state levels of identity fusion among participants who reported high negative affect.

**Table 2:** The effect of 2013 Boston Marathon Bomb prime on DIFI.

Negative affect	Effect	SE	*p*	LLCI	ULCI
−1.9671	−7.987	7.9487	.3205	−23.7189	7.8416
.0000	2.6542	4.5700	.5628	−6.4185	11.7269
−1.9671	13.2471	5.4421	.0168*	2.4432	24.0510

#### Discussion

Study 3 explored the hypothesis that shared negative experience is a causal antecedent of identity fusion from a slightly different angle from Study 1 and Study 2. Whereas the first two studies adopted a cross-sectional correlational approach to examine the process by which exposure to shared negative experience increased trait levels of fusion via the chronic accessibility of the negative experiences, Study 3 adopted an experimental approach to examine how temporarily increasing the accessibility of negative experiences affects state levels of fusion. Although there were no main effects of the prime on state fusion on either measure of state fusion (viz., DIFI, verbal fusion scale), increased salience of the shared negative event—the 2013 Boston Marathon Bombing—did elevate levels of state fusion among individuals who experienced high levels of negative affect. Thus, consistent with Study 1 and Study 2, Study 3 provides evidence that increased accessibility of shared negative experience enhances identity fusion among group members.

## General Discussion

Across three studies—two correlational, and one experimental—we investigated the role of shared negative experience in enhancing identity fusion among group members. In the first study, we found that Northern Irish Republicans and Unionists who had experienced more shared negative experience (i.e., suffering as members of their respective groups) also reflected more about their experiences; furthermore, those who reflected more about their experiences were in turn more fused with their groups. However, the evidence for our causal model was incomplete in this case, as an alternative simple mediation model similarly fitted the data.

In the second study, we found that Bostonians who had experienced the 2013 Boston Marathon Bombing more directly or who had suffered more as a result of that tragedy also reflected more about the event; furthermore, those who reflected more were in turn more fused with their groups. This time, the alternative causal model enjoyed less theoretical plausibility, and simple mediation analyses showed relatively weak effects. As no correlational study can provide direct evidence for a causal model, a third study was run. This study aimed to resolve the causal ambiguity of the first two correlational studies by investigating the effects of increased temporary cognitive accessibility of a shared negative event on state levels of identity fusion. While there was no main effect of the priming manipulation on fusion, increased salience of the 2013 Boston Marathon Bombing did elevate state levels of fusion among individuals whose affective responses were more negative.

Taken together, these three studies provide some early evidence for the claim that shared negative experience—particularly, shared negative experience that leads to reflection—is a causal antecedent to identity fusion [7, 28–30], which previous research has shown to be an important and unique predictor of costly pro-group behaviour. Some questions remain, however. For example, the precise nature of the personal reflection that joins shared negative experience to identity fusion is still unclear. Due to the limitations on study duration imposed by our method of data collection, we were unable to probe into precisely how individuals reflected or ruminated upon the events in question. Indeed, our measures of reflection were rather brief and *ad hoc* constructions for the purposes of the present research. Future research will need to employ psychometrically-validated and theoretically-driven measures to elucidate whether, for example, different kinds of self-attentiveness (e.g., rumination or *neurotic* self-attentiveness; reflection or *intellectual* self-attentiveness [31]) feature differently in the relationship between shared negative experience and identity fusion. Similarly, while affective responses were measures in Study 3, Studies 1 and 2 left the extent to which negative affect influences the relationship between shared negative experience and identity fusion unexplored.

Previous research on the consequences of identity fusion has shown that high levels of fusion can lead to increased endorsement of extreme pro-group behaviours, particular when the groups in question are threatened. In our studies on the Northern Irish conflict and the Boston Marathon Bombing, we see how experiencing shared negative experiences at the hands of hostile outgroups fuses people with their groups. This and further understanding of the process by which cycles of intergroup aggression are perpetuated are important if we are to develop effective interventions to stem the tide of seemingly intractable conflicts around the world. Our findings about the relationships between shared negative experience, reflection and fusion might suggest various junctures at which to obstruct pathways to violence. For example, they might suggest that reframing negative experience as individual rather than shared might reduce the effect of negative experience on identity fusion. They might also suggest that minimising reflection or rumination—particularly the kind of reflection that leads to interpreting events as self-defining—would be beneficial. These potential interventions require further exploration and empirical evaluation, in light of the present research.

## Supporting Information

S1 File Additional instructions, measures, and materials. (DOCX)

S2 File Raw data for Study 1. (XLSX)

S3 File Raw data for Study 2. (XLS)

S4 File Raw Data for Study 3. (XLSX)
