# Supplementary material for: Shared Negative Experiences Lead to Identity Fusion via Personal Reflection
Source: PLoS One. 2015 Dec 23;10(12):e0145611. doi: 10.1371/journal.pone.0145611 (PMC4689389; doi:10.1371/journal.pone.0145611)
Supplement: S1 File — (DOCX) [file pone.0145611.s001.docx]

This document contains the instructions, manipulations, and measures that were not included in the main text.

**Study 1**

Shared trauma measure

We would like to know more about your experiences as a Republican^^[[1]](#footnote-1)^^ in Northern Ireland. Please indicate whether you have had the following experiences: Y/N

Have you been physically attacked or injured for being a Republican?

Have you been verbally attacked for being a Republican?

Have you been publicly humiliated for being a Republican?

Have your home or property been vandalised or stolen for being a Republican?

Have any of your friends and family been physically attacked or injured for being Republicans?

Have you witnessed your friends and family been physically attacked or injured for being Republicans?

Have any of your friends and family been verbally attacked for being Republicans?

Have you witnessed your friends and family been verbally attacked for being Republicans?

Have any of your friends and family been publicly humiliated for being Republicans?

Have you witnessed your friends and family been publicly humiliated for being Republicans?

Have the home or property of your family been vandalised or stolen for being Republicans?

Have you witnessed the home or property of your family been vandalised or stolen for being Republicans?

Identity fusion measure

Below are a list of statements about your relationship to Republicanism^^[[2]](#footnote-2)^^.

Please read the statements and indicate on the scale to what extent you would agree with the statements on the left.

Strongly Disagree—Disagree—Somewhat Disagree—Somewhat Agree—Agree—Strongly Agree

I am one with Republicanism.

I feel immersed in Republicanism.

I have a deep emotional bond with Republicanism.

Republicanism is me.

I'll do for Republicanism more than any other Republicans would.

I am strong because of Republicanism.

I make Republicanism strong.

**Study 2**

Boston Bombing Salience Task

The following few questions are about the Boston Marathon Bombings that took place in April 2013. First, you will be asked to recall as vividly as possible your experience of the events. Then, you will be asked specific questions about where you were and how felt at the time.

Please click Continue when you are ready to proceed.

We would now like you to recall--as vividly as you can--the Boston Marathon Bombings that took place in April 2013. What happened at the bombing? Where were you when it happened? Did it affect you or anyone you know directly? How did it make you feel? Please write down your recollections of the event and your experiences in the space provided below:

**Study 3**

Boston Bombing Salience Condition

We would now like you to recall--as vividly as you can--the Boston Marathon Bombings that took place in April 2013. What happened at the bombing? Where were you when it happened? Did it affect you or anyone you know directly? How did it make you feel? Please write down your recollections of the event and your experiences in the space provided below:

Control Condition

We would now like you to recall--as vividly as you can--a recent experience of running errands in Boston, doing commonplace things such as shopping for groceries. What were the errands in question? Where did you go? Did you accomplish your goals? What was the commute like? How did it make you feel? Please write down your recollections of the event and your experiences in the space provided below:

Semantic differential measure of core affect

Please use the scales below to indicate how thinking about the event made you feel:

Each scale on this page contains an adjective pair which you will use to rate how you currently feel. Some of the pairs may seem unusual, but you will probably feel more one way than another. So, for each pair,click the button closest to the adjective which you believe describes your current feelings better. The more appropriate the adjective seems, the closer the button you click should be.

Thinking about the event made me feel:

Unsatisfied O O O O O O O O O Satisfied

Controlled O O O O O O O O O Controlling

Dull O O O O O O O O O Jittery

Sleepy O O O O O O O O O Wide awake

Melancholic O O O O O O O O O Contented

Annoyed O O O O O O O O O Pleased

Awed O O O O O O O O O Important

Unhappy O O O O O O O O O Happy

Influenced O O O O O O O O O Influential

Despairing O O O O O O O O O Hopeful

Cared for O O O O O O O O O In control

Bored O O O O O O O O O Relaxed

Submissive O O O O O O O O O Dominant

Unaroused O O O O O O O O O Aroused

Sluggish O O O O O O O O O Frenzied

Relaxed O O O O O O O O O Stimulated

Calm O O O O O O O O O Excited

Guided O O O O O O O O O Autonomous

DIFI (see <http://www.uned.es/fusion/difi/>)

We are interested in how you feel right now toward Boston.

The diagram below is designed to represent your relationship with Boston.

Please indicate your relationship by clicking and dragging the smaller "Me" circle to the position that best captures your current relationship with this group.

1. Or “Unionist”; *mutatis mutandis* below. [↑](#footnote-ref-1)
2. Or “Unionism”; *mutatis mutandis* below. [↑](#footnote-ref-2)
